# Stimulating endogenous cardiac repair

**DOI:** 10.3389/fcell.2015.00057

**Published:** 2015-09-29

**Authors:** Amanda Finan, Sylvain Richard

**Affiliations:** Centre National de la Recherche Scientifique United Medical Resource 9214, Institut National de la Santé et de la Recherche Médicale U1046, Physiology and Experimental Medicine of the Heart and Muscles, University of MontpellierMontpellier, France

**Keywords:** cell therapy, cardiac progenitor cells, regeneration, gene therapy, stem cells, beta blockers, statins

## Abstract

The healthy adult heart has a low turnover of cardiac myocytes. The renewal capacity, however, is augmented after cardiac injury. Participants in cardiac regeneration include cardiac myocytes themselves, cardiac progenitor cells, and peripheral stem cells, particularly from the bone marrow compartment. Cardiac progenitor cells and bone marrow stem cells are augmented after cardiac injury, migrate to the myocardium, and support regeneration. Depletion studies of these populations have demonstrated their necessary role in cardiac repair. However, the potential of these cells to completely regenerate the heart is limited. Efforts are now being focused on ways to augment these natural pathways to improve cardiac healing, primarily after ischemic injury but in other cardiac pathologies as well. Cell and gene therapy or pharmacological interventions are proposed mechanisms. Cell therapy has demonstrated modest results and has passed into clinical trials. However, the beneficial effects of cell therapy have primarily been their ability to produce paracrine effects on the cardiac tissue and recruit endogenous stem cell populations as opposed to direct cardiac regeneration. Gene therapy efforts have focused on prolonging or reactivating natural signaling pathways. Positive results have been demonstrated to activate the endogenous stem cell populations and are currently being tested in clinical trials. A potential new avenue may be to refine pharmacological treatments that are currently in place in the clinic. Evidence is mounting that drugs such as statins or beta blockers may alter endogenous stem cell activity. Understanding the effects of these drugs on stem cell repair while keeping in mind their primary function may strike a balance in myocardial healing. To maximize endogenous cardiac regeneration, a combination of these approaches could ameliorate the overall repair process to incorporate the participation of multiple cellular players.

## Introduction

Despite the significant research effort that has gone into improving therapeutics and interventions for coronary heart disease, it still remains a great societal, economic and clinical burden throughout the world. In particular, myocardial infarction is attributable to more than half of the cardiovascular disease related mortality in the industrialized world (Go et al., 2014). The coronary occlusion in a myocardial infarct results in significant cardiac cell damage and death. Current therapies are able to attenuate symptoms and prolong life. However, an intervention has not yet been developed that can replace or regenerate the damaged myocardium after an infarct. This is due in part to the absence of understanding and the potential of how the heart tissue can be repaired or regenerated.

**Figure 1:**
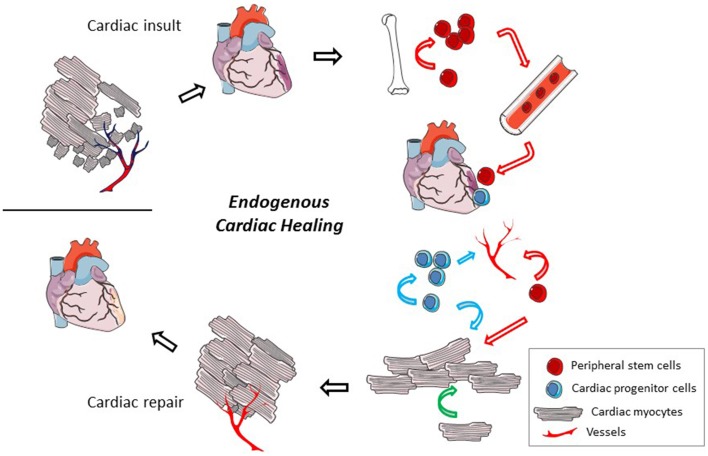
**Endogenous cardiac regeneration**. After a cardiac insult, a significant number of cardiac mycoytes die and vessel density gets reduced. In a very limited way, the heart has the ability to regenerate but it is insufficient to compensate for the total damage. The cellular participants in the endogenous regeneration process may include cardiac myocytes (pink cells, black lines), local cardiac progenitor cells (blue cells), and recruited peripheral stem cells (red cells). These cells have the potential to proliferate and participate in the regeneration of cardiac myocytes, angiogenesis, and the release of trophic factors that may reduce cardiac cell death. Modes of the cells participation are identified by colored arrows (peripheral stem cell, red; cardiac progenitor cell, blue; cardiac myocyte, green). Methods that could be employed to improve these endogenous mechanisms include cell and/or gene therapy and pharmacologic treatments.

Evidence has accumulated over the past 15 years to demonstrate that there is some amount of cardiac turnover, both in healthy and injured tissue. Particular interest has focused on cardiac regeneration after ischemic events due to the large amount of myocyte death, but reports also describe cardiac proliferation in models of cardiac pressure overload and idiopathic dilated cardiomyopathy (Kajstura et al., 1998; Urbanek et al., 2003). Cardiac cell proliferation is highest in youth, reducing with increased age (Rumyantsev and Borisov, 1987; Mollova et al., 2013; Naqvi et al., 2014). In an average healthy adult, the cardiomyocyte turnover rate is controversial with reports predicting annual turnover rates ranging anywhere from 0.3% to more than 50% (Bergmann et al., 2009; Kajstura et al., 2010; Bergmann and Jovinge, 2014). Methods have been questioned in studies where turnover is on the higher end (Laflamme and Murry, 2011). Cardiomyocyte renewal is augmented in cardiac pathological settings such as myocardial infarction and heart failure (Rumyantsev and Borisov, 1987; Kajstura et al., 1998; Beltrami et al., 2001). Due to these evidences, it appears that the heart has some capacity for renewal though it is limited and unable to compensate for significant cardiac damage. It has been proposed that evolutionary mechanisms have limited pathways of cardiac regeneration to protect the heart from developing cancer (Penn, 2010). In fact, primary cardiac tumors are extremely rare, representing only 0.3–0.7% of all cardiac tumors (Leja et al., 2011). This endogenous capability to regenerate does provoke a potential target for cardiac therapies however. Reports demonstrate multiple cellular players participating in endogenous cardiac regeneration (Figure 1). The exact contribution of each cell type remains a topic of controversy in the cardiac regenerative medicine field. Focusing the repair potential to just one cell type may actually limit the therapeutic opportunities. Instead it is likely that provoking each of these players could provide an orchestrated and more complete regeneration.

## Participants in cardiac regeneration

### Cardiac myocytes

Amputation studies in zebrafish and neonatal mice brought to light the potential for cardiac regeneration by cardiac myocytes themselves (Poss et al., 2002; Porrello et al., 2011). The mechanisms proposed include the activation of cardiogenesis and cell cycle genes and dedifferentiation (Jopling et al., 2010; Kikuchi et al., 2010; Porrello et al., 2011; Mahmoud et al., 2013). However, this capacity for complete regeneration in the mouse is lost by 1 week of age (Porrello et al., 2011). Studies are incongruous on whether cardiac myocyte proliferation continues into adult life (Walsh et al., 2010; Senyo et al., 2013). It does appear likely that myocardial injury can induce to some extent pre-existing cardiac myocyte renewal (Senyo et al., 2013).

### Cardiac progenitor cells

Cardiac progenitor cells (CPC) are an adult stem cell population residing in the heart. These cells have been identified by a number of markers including c-kit, sca-1, Isl1, Wilms tumor 1, and the ability to form cardiospheres *in vitro* (Beltrami et al., 2003; Genead et al., 2010; Smart et al., 2011; Hsiao et al., 2014; Uchida et al., 2013). It is evident that after a cardiac injury these cell types proliferate and increase in number (Fransioli et al., 2008; Smart et al., 2011; Uchida et al., 2013). It has even been suggested that these cells are indispensable for cardiac regeneration (Ellison et al., 2013). Disagreement exists on the exact differentiation capabilities of these cells *in vivo*, particularly with the c-kit^+^ CPC population. Research consistently reports the ability of the cells to differentiate into an endothelial cell lineage but their contribution to cardiac myocytes has been questioned (Hsieh et al., 2007; Fransioli et al., 2008; Jesty et al., 2012). Ellison et al. found that c-kit^+^ CPC significantly contribute to *de novo* cardiac myocyte generation; whereas van Berlo et al. reported that differentiation of c-kit^+^ cells to cardiac myocytes is negligible (Ellison et al., 2013; Van Berlo et al., 2014). This discrepancy may in part be due to the different transgenic mouse lines used between the two studies. A study with the same mouse line used in the Ellison study demonstrated that of the c-kit^+^ cells identified by immunofluorescence, only 32% of the cells were positive for fluorescent protein driven by the c-kit promoter (Fransioli et al., 2008). This suggests that the results of the Ellison study may potentially underestimate the involvement of c-kit^+^ cells. Regardless of these issues, it is evident that CPC are responsive to cardiac injury and differentiate into endothelial cells, aiding in increased vessel density, a mechanism correlated to improved cardiac function (Kim et al., 2014).

### Peripheral stem cells

The bone marrow is a reservoir for multiple populations of adult stem cells including, but not limited to, endothelial progenitor cells (EPC), c-kit^+^ stem cells, very small embryonic like stem cells, mesenchymal stem cells (MSC), and hematopoietic stem cells. Bone marrow cells are mobilized into the peripheral blood after cardiac injury and are recruited to the myocardium (Mouquet et al., 2005; Finan et al., 2012). The recruitment of bone marrow cells is associated with cardiac repair and along the same lines, in studies where the bone marrow stem cells response is attenuated, reduced accumulation of bone marrow cells is associated with worse outcomes (Fazel, 2006; Sopko et al., 2010; Finan et al., 2013; Gong et al., 2014). Bone marrow stem cells can incorporate into the vasculature, differentiate into endothelial cells, and participate in vascular remodeling (Finan et al., 2013; Wu et al., 2015). Studies of gender mismatched bone marrow and heart transplant patients demonstrated that bone marrow cells have the capacity to differentiate into cardiac myocytes (Quaini et al., 2002; Deb et al., 2003). However, a recent study suggests that bone marrow cells do not directly differentiate into cardiac myocytes and instead suggests their fusion with existing cardiac myocytes (Wu et al., 2015). Bone marrow cells may also replenish the diminished CPC pool after a cardiac injury (Mouquet et al., 2005; Barile et al., 2011).

## Improvement of endogenous cardiac healing

It is clear that the heart has some capacity to regenerate through various cell participants to support normal deterioration. Unfortunately, it is evident that the potential is limited and unable to compensate for the extent of damage that may occur after a myocardial infarction. A number of approaches have been attempted to improve cardiac regeneration, including cell and gene therapy. Reasonable benefits have been determined from these therapeutic approaches though the beneficial mechanisms lie in the trophic factors produced or through the activation of endogenous cardiac healing. Recently, it is has been identified that common drugs administered to patients with cardiovascular disease may alter endogenous stem cell populations. Profiting from these interactions may be a potential manner to assist in cardiac repair.

### Cell therapy

Attempts to repair the damaged myocardium with cardiac myocytes or skeletal myocytes have shown limited potential. A large amount of enthusiasm therefore has focused on the potential that stem cells could have in cardiac regenerative therapy. Many stem cell types have been examined including embryonic stem cells, induced pluripotent stem cells, various bone marrow stem cell types, and cardiac stem cells. Cell therapy efforts have been reviewed more exhaustively elsewhere (Goumans et al., 2014; Pavo et al., 2014).

Embryonic stem cells (ESC) and induced pluripotent stem cells have a great power to differentiate into all cell types making them an attractive choice for replacing lost myocardium. While efforts have been made to program these stem cell types to reduce tumor potential, it still remains a risk as well as a potential immunological challenge in the recipient (Anderson et al., 2014; De Almeida et al., 2014; Zhang et al., 2015). Cardiac myocytes derived from human pluripotent stem cells were shown to be anti-arrhythmic in a guinea pig model (Shiba et al., 2012). However, in a large primate model, the transplantation of human ESC derived cardiac myocytes was associated with ventricular arrhythmias, although they were transient and did not induce sudden death (Chong et al., 2014). As the human heart has a significantly slower heart rate and larger size than the primate model, the propensity of arrhythmias from ESC transplant increases (Lalit et al., 2014). For this reason, the transplantation of ESC derived cardiac myocytes needs to be tested with caution in humans (Anderson et al., 2014).

**Figure 2:**
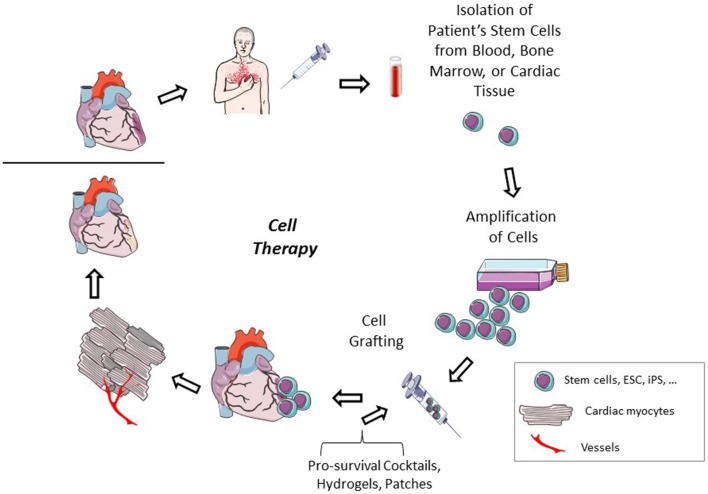
**Cardiac cell therapy**. After a cardiac injury, a patient's adult stem cells are isolated. These cells can be isolated from either cardiac tissue or from peripheral sites. The cells are amplified to have a sufficient number for therapeutic transplant. The cells are injected into the patient either intravenously or intracardiac, alone or in the presence of pro-survival cocktails, hydrogels, or extracellular matrix patches. The injected cells can participate themselves in cardiac regeneration or improve endogenous healing by excreting paracrine factors which reduce apoptosis and increase stem cell recruitment and activity and angiogenesis to repair the damaged cardiac tissue.

Due to the issues that still remain with pluripotent stem cells, adult stem cells could be a more plausible option for cell therapy. Autologous adult stem cells can be delivered to patients reducing any risk for immune complications. As these cell types have a more differentiated lineage, no concerns for tumor formation remain. In animal models, cardiac cell therapy with bone marrow stem cells or cardiac stem cells can improve cardiac function, decrease infarct size, improve myocardial regeneration, increase vascular density, and activate endogenous stem cell populations (Figure 2; Yoon et al., 2010; Loffredo et al., 2011; Dong et al., 2012; Avolio et al., 2015). These benefits seem to translate into similar results in a portion of human clinical trials. Individual reports have described improved cardiac function, decreased scar size, and improved physical capabilities, such as improvements in the 6 min walking distance test, after adult stem cell therapy (Bolli et al., 2011; Malliaras et al., 2014b; Golpanian et al., 2015; Mathiasen et al., 2015). However, results from the meta-analysis of cell based cardiac studies revealed no clinical benefit 3–12 months after cell therapy (Gyöngyösi et al., 2015).

The beneficial mechanism of adult stem cells has not been direct differentiation to cardiac myocytes as originally hoped for. Instead the more prominent mechanism appears to involve the activation of endogenous healing pathways through paracrine factors provided by the transplanted cells. These pathways can reduce myocardial apoptosis, activate cardiac myocytes proliferation and endogenous stem cell recruitment (Zhang et al., 2007; Fischer et al., 2009; Malliaras et al., 2014a; Avolio et al., 2015). Cell therapy also aids in improving vascular density in the damaged myocardium either through direct differentiation or activating endogenous angiogenesis. The importance of this mechanism to cardiac repair was highlighted recently (Yoon et al., 2010). In this study, infarcted mice were treated with ganciclovir after MSC therapy to induce cytotoxicity in stem cell derived endothelial cells. The death of the endothelial cells and the subsequent decrease in vessel density led to a significant deterioration of cardiac function. Even though cell therapy approaches may not themselves replace the lost cardiac myocytes, their release of trophic factors that reduce apoptosis or recruit endogenous stem cells and their ability to aid in vascular remodeling may provide a significant benefit to cardiac repair.

An additional point to note is that the retention of the stem cells in the myocardium is extremely low, reducing their potential therapeutic effects. The transplantation of sheets of cells or in combination with hydrogels or patches improve the cell engraftment rate, thus improving cardiac function (Ishii et al., 2014; Roche et al., 2014; Tano et al., 2014; Zhang et al., 2015). Interestingly, even altering the growth media of the cells can improve their retention in the tissue (Kim et al., 2014). Pro-survival cocktails can also be used to improve the duration and number of the cells in the myocardium (Laflamme et al., 2007; Gautam et al., 2015). Components of the pro-survival cocktails include growth factors, anti-apoptotic molecules such as cell-permeant Bcl-XL and caspase inhibitors, immunosuppressants, and vasodilators.

### Gene therapy

Natural reparative signaling pathways occur in the heart after a cardiac injury. However, these signals are usually transient, not enduring long enough to support complete endogenous repair (Askari et al., 2003; Schenk et al., 2007). As discussed in the previous section, transplanted stem cells provide paracrine factors that support natural tissue repair. Gene therapy is a mechanism to capitalize on these natural factors to prolong or reactivate the signals. Cardiac diseases gene therapy has encompassed delivery options including adenovirus, direct plasmid transfer, microbubbles and modified RNA.

**Figure 3:**
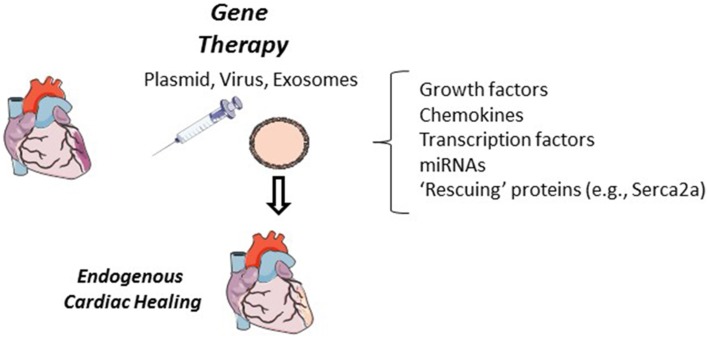
**Cardiac gene therapy**. In animal models, cardiac gene therapy has shown to be successful when delivered by direct plasmid, in a lenti- or adenovirus, or in exosomes. Growth factors, chemokines, transcription factors and miRNAs have all been tested in cardiac gene therapy. Expression of these proteins and miRNAs have the potential to improve endogenous cardiac regeneration through the recruitment, activation and differentiation of local and peripheral stem cells, improved vascular density, or by proliferation of cardiac myocytes. Additional cardiac “rescuing” gene therapy targets (e.g., Serca2a) can improve cardiac function but it has yet to been investigated if these approaches can alter endogenous cardiac regeneration.

Growth factors and chemokines are greatly implicated in cardiac repair and their expression through gene therapy techniques can improve cardiac function (Kanashiro-Takeuchi et al., 2011). Beneficial mechanisms induced by this approach include elevated proliferation, mobilization, and recruitment of endogenous local and peripheral stem cell populations (Figure 3). A number of growth factor or cytokine candidates have been demonstrated to be favorable in cardiac repair, including, but not limited to, vascular endothelial growth factor, monocyte chemotactic protein 3, and placental growth factor (Schenk et al., 2007; Iwasaki et al., 2011; Zangi et al., 2013). In particular, stromal derived factor-1 expression can recruit various C-X-C chemokine receptor type 4 positive cell populations including CPC, MSC and very small embryonic like stem cells (Askari et al., 2003; Kucia et al., 2006; Dong et al., 2012). Stem cell factor gene therapy has been described to induce cardiac myocyte and CPC proliferation (Yaniz-Galende et al., 2012; Ishikawa et al., 2015). Gene therapy by direct injection of growth factor or chemokine plasmids, namely stromal derived factor-1 and vascular endothelial growth factor, has been demonstrated in patients with ischemic heart disease to be safe and show signs of a functional benefit (Favaloro et al., 2013; Penn et al., 2013). In particular, stromal derived factor-1 plasmid injection into cardiac heart failure patients has shown functional therapeutic promise by improving ventricular remodeling in patients with more advanced cardiac dysfunction (Chung et al., 2015).

Transcription factors have also been targeted in cardiac gene therapy. Hypoxia inducible factor 1-alpha has been an appealing target as it is protective by regulating angiogenesis and survival, as well as the expression of chemokines capable of recruiting stem cells (Semenza, 2014). Plasmid or adenovirus transfer of hypoxia inducible factor 1-alpha into the myocardium can improve cardiac repair, decrease cardiac myocyte apoptosis, mobilize bone marrow stem cells to the heart, and activate c-kit^+^ cells in the myocardium (Huang et al., 2011, 2014; Ong et al., 2014). *In vivo* reprogramming of fibroblasts with the cardiac transcription factors GATA4, Mef2c, and Tbx5 generates functional cardiac myocytes which are fully incorporated in the myocardium and decrease scar size, thus improving cardiac function (Inagawa et al., 2012; Qian et al., 2012). An additional tested approach is the manipulation directly of the proliferation of the cardiac myocytes. Injection of an adenovirus expressing cardiac specific telomerase or the Yes-associated protein can induce cardiac myocyte proliferation and improve overall cardiac repair (Bär et al., 2014; Lin et al., 2014).

MicroRNAs (miRNAs) have also been employed in gene therapy techniques to promote cardiac regeneration. miRNAs are small non-coding RNAs that bind mRNA and inhibit translation. Eulalio et al. performed a high throughput screening of miRNAs on neonatal rat cardiac myocytes to identify candidates that induce proliferation (Eulalio et al., 2012). They then demonstrated that in fact when an adenovirus expressing either miR-590-3p or miR-199a was injected at the time of myocardial infarction, they could induce cardiac myocyte proliferation resulting in decreased infarct size and improved cardiac function. Endogenous CPC proliferation was elevated after lentiviral delivery of miR-17-92 (Sirish et al., 2012). The *in vivo* reprogramming of cardiac fibroblasts to cardiac myocytes by lentiviral transfer of a combination of miRNAs (1, 133, 208, 499) has also been demonstrated to be a successful approach to induce endogenous cardiac regeneration (Jayawardena et al., 2015). miRNAs can also be a target of inhibition. To combat the anti-angiogenic properties of miR-92a, an antagomir was designed to inhibit the miRNA thus increasing capillary density after myocardial infarction (Bonauer et al., 2009). The potential of miRNAs in cardiac repair has been reviewed more thoroughly elsewhere (Hodgkinson et al., 2015).

Additional targets of cardiac gene therapy have included calcium cycling proteins, extracellular matrix alterations, adrenergic manipulation, and arrhythmias and have been reviewed elsewhere (Nagai and Komuro, 2012; Scimia et al., 2013). These approaches appear to have a sufficient effect on their target and improve cardiac function but besides the mention that targeting calcium cycling proteins can block cardiac remodeling, little is known on the effect of these strategies and the survival or regeneration of the tissue (Katz et al., 2014). It would be interesting to examine the cell proliferation or anti-apoptotic effects of these therapies especially as atrial fibrillation and changes in calcium channels and intracellular calcium changes can alter cardiac myocyte and CPC proliferation (Ferreira-Martins et al., 2009; Chernyavskaya et al., 2012; Gambini et al., 2012; Demion et al., 2014).

### Pharmacological interventions

The common pharmaceutics given to patients who have had a myocardial infarction include aspirin, beta blockers, ace inhibitors, angiotensin receptor blockers, and statins. These drugs aim at reducing blood clots, regulating blood pressure, stabilizing electrical activity, and reducing cholesterol. These approaches improve patient lifestyle and prolong life but they do not replace the damaged tissue that is lost after a myocardial infarction. However, evidence is mounting that these drugs may actually have either positive or negative effects on endogenous stem cell populations and cardiac myocyte survival and proliferation (Table 1). Potential may exist in refining current therapies to control their primary functions and generate a balance in myocardial healing. These drug regiments, as well as, patient's age and pathophysiological state likely have grave implications on endogenous stem cell activity as well as exogenous cell therapy approaches. Increased age and cardiovascular comorbidities have well been described to impair endogenous cardiac repair (Gambini et al., 2012; Lee and Poh, 2014; Hariharan and Sussman, 2015). Research regarding the effects of these parameters has been limited in animal studies. Awareness of these effects will allow clinicians to anticipate potential adverse effects and facilitate maximal results.

**Table 1:** **Effects of common pharmaceuticals on cardiac myocytes and endogenous stem cells**.

	**Aspirin**	**Beta Blockers**		**ACE inhibitors/angiotensin receptor blockers**	**Statins**
		**Beta 1**	**Beta 2**		
Cardiac myocytes	↓ Viability	↓ Apoptosis	↑ Apoptosis	↓ Apoptosis	↓ Apoptosis
	Tan et al., 2013	↓ Inotropy	↓ Cardioprotection	Kajstura et al., 1997; Xu et al., 2010	↑ Proliferation
		Bristow et al., 1986; Seqqat et al., 2012	Zhang et al., 2010		Ito et al., 2004; Tang et al., 2011; Zhang et al., 2012
Cardiac progenitor cells	?	↓ Apoptosis	↓ Proliferation		↑ Number
		Khan et al., 2013	Ellison et al., 2007		↑ Proliferation
					Suzuki et al., 2009; Gambini et al., 2012
Peripheral stem cells	↓ Number	↑ Number	?	↑/↓ Number> (?)	↑ Number
	↓ Migration	Sorrentino et al., 2011		↑/↓ Differentiation (?)	↑ Mobilization
	↓ Proliferation			Thum et al., 2011; Durik et al., 2012; Jung et al., 2012; Li et al., 2012; Wu et al., 2013; Liu et al., 2015	↑ Proliferation
	↑ Apoptosis				↑ Survival
	Deng et al., 2009; Boer et al., 2011; Sato et al., 2013				↑ Differentiation
					Erbs et al., 2011; Shaw et al., 2011; Cai et al., 2013; Cohen et al., 2013

The common pharmaceuticals prescribed after a cardiac injury are detailed. The positive effects on cardiac myocytes, cardiac progenitor cells, or peripheral stem cells are highlighted in blue and negative effects are highlighted in red. Blockade of the beta adrenergic signaling system has been divided by receptors beta 1 and beta 2.

#### Aspirin

Aspirin can reduce the risk of myocardial infarction in high-risk patients and prevent an additional event. However, contradictions exist with aspirin including an increased risk of gastrointestinal bleeding. Chronic treatment with aspirin may inhibit the cardiac proteasome and reduce cardiac myocyte viability (Tan et al., 2013). Interestingly, aspirin may have negative effects on endogenous stem cell populations as well. Aspirin can reduce EPC number and migration capacity (Boer et al., 2011; Sato et al., 2013). Aspirin may also inhibit MSC proliferation and induce apoptosis (Deng et al., 2009). No reports have yet described the effects of aspirin on CPC but as non-steroidal anti-inflammatory drugs can negatively affect ESC cardiac differentiation, it could have an implication on *in vivo* cardiac differentiation as well (Chillar et al., 2011). The negative effects of aspirin on endogenous stem cells may be due to its anti-inflammatory effects. Pro-inflammatory signals have been demonstrated to aid in regulating and activating endogenous stem cell populations (Reber et al., 2006; Mirantes et al., 2014).

#### Beta blockers

After a myocardial infarction, an excessive number of catecholamines are released. To reduce their deleterious action, beta adrenergic receptor blocking agents are administered to patients. These drugs aid in the prevention of additional infarcts and reduce cardiac arrhythmias. The majority of the inotropic effects of the beta adrenergic signaling pathway are mediated by the beta 1 receptor (Bristow et al., 1986). This pathway as well can promote cardiac myocytes apoptosis (Seqqat et al., 2012). Conversely, beta 2 adrenergic receptor signaling is anti-apoptotic and cardioprotective (Zhang et al., 2010). Interestingly, general blockade of beta adrenergic receptor signaling by propanolol in cardiac pressure overload actually induces cell cycle gene expression and increases the number of ki-67 proliferating cardiac myocytes primarily through the beta 2 receptor pathway (Musumeci et al., 2011).

Catecholamines can stimulate proliferation in endogenous CPC through beta 2 adrenergic receptor signaling which is the more highly expressed beta adrenergic receptor on these cells (Ellison et al., 2007). Similarly to cardiac myocytes, stimulation of beta 1 adrenergic receptor can induce CPC apoptosis (Khan et al., 2013). Remarkably, a higher number of isolated CPC has been associated with patients on beta blocker therapy (Gambini et al., 2012). Beta adrenergic signaling manipulation may also alter peripheral stem cell activity. The beta 1 specific adrenergic receptor blocker, nebivolol, can increase EPC number after myocardial infarction (Sorrentino et al., 2011). Clenbuterol, a beta 2 receptor agonist, along with the chemokine GCSF, augments peripheral blood CD34^+^ cells after myocardial infarction (Tanaka et al., 2013). Research is scarce on the beta adrenergic receptor signaling role in endogenous stem cell differentiation. As activation of this pathway can improve cardiac differentiation in ESC, it may be interesting in future studies to examine CPC and peripheral stem cell differentiation after manipulation of this pathway (Yan et al., 2011).

Clearly manipulation of the beta adrenergic receptor pathway may improve endogenous cardiac regeneration. Stimulating the beta 2 receptor could likely reduce cardiac myocytes apoptosis and improve the local and peripheral endogenous stem cell reservoir. It is likely that treatment with a beta 1 receptor specific blocker and a beta 2 specific agonist may provide a balance in cardiac therapy as recently demonstrated (Sun et al., 2014).

#### Angiotensin converting enzyme inhibitors/angiotensin receptor blockers

The renin/angiotensin aldosterone system (RAAS) is altered in cardiovascular diseases. In heart failure and after a myocardial infarction, drugs (e.g., angiotensin converting enzyme inhibitors, angiotensin receptor angatonists) which block this pathway are recommended to relax blood vessels, thereby reducing blood pressure (Mentz et al., 2013). These drugs may aid in reducing apoptosis of cardiac myocytes as well. Angiotensin II signaling can increase apoptosis of cardiac myocytes (Kajstura et al., 1997; Xu et al., 2010). Conversely, the benefits of these drugs are unclear for stem cells. For example, one report described that the activation of angiotensin like-1 receptor after myocardial infarction increased the accumulation of vascular stem cells, while another article found instead that blockade of the signaling increases the number of EPC (Jung et al., 2012; Li et al., 2012). Opposing results also exist on the angiogenic properties of EPC in the presence of RAAS signaling (Thum et al., 2011; Durik et al., 2012). The RAAS may actually be beneficial for other stem cell types; improving cardiac differentiation of ESC and MSC and rendering MSC more apoptotic resistant and amplifying their growth factor expression (Durik et al., 2012; Wu et al., 2013; Liu et al., 2015). Little has been investigated on the RAAS and CPC. The only article published to date identifies a specific angiotensin 1 receptor expressing CPC population that is senescent with impaired growth and an increased susceptibility to apoptosis (D'Amario et al., 2011). Overall, the use of drugs antagonizing the RAAS are beneficial toward cardiac function and cardiac myocyte survival, but their use in stem cell mediated regeneration requires a further, more detailed investigation.

#### Statins

Statins are cholesterol lowering drugs and have also been associated with having anti-inflammatory and anti-thrombotic effects (Angeli et al., 2012). Statin treatment has been described to block apoptosis of cardiac myocytes and can elevate the number of proliferating cardiac myocytes after a myocardial infarct (Ito et al., 2004; Suzuki et al., 2009; Tang et al., 2011; Zhang et al., 2012). Statins also have positive effects on endogenous stem cell populations. Progenitor cells in the heart are elevated after statin treatment and myocardial infarction (Suzuki et al., 2009). The amplification potential of CPC is significantly higher from patients on statins (Gambini et al., 2012). Bone marrow stem cells and EPC are also positively regulated by statins. Elevated number, mobilization, proliferation, improved differentiation capacity, and increases in survival have all been described in peripheral stem cell populations after statin treatment (Erbs et al., 2011; Shaw et al., 2011; Cai et al., 2013; Cohen et al., 2013). Taken all together, statin therapy appears to enhance endogenous cardiac repair pathways.

#### Additional approaches

A few novel approaches have been described in recent years to improve endogenous stem cell mediated cardiac regeneration. A unique idea proposed was to find a method to trap the circulating stem cells to the tissue where their action is required. Baumer et al. generated a bi-functional fusion protein that binds damaged vessels and CD133^+^ progenitor cells (Baumer et al., 2012). They were able to demonstrate that this molecule could improve progenitor cell adhesion thereby increasing capillary density and improving cardiac function. Tetravalent antibodies have also been employed for the same goal. These antibodies were generated to target an epitope on apoptotic cardiac myocytes, CD34, CD133, and c-kit cells (Malecki et al., 2013). This approach significantly improved the recruitment and retention of the various stem cell populations to the damaged myocardium.

As described above, stem cell populations provide trophic factors to the damaged myocardium. Due to this idea, exosomes have been exploited as a possible means to improve cardiac regeneration. In fact, ESC exosome delivery after a myocardial infarct improved overall cardiac function, increased capillary density and proliferating cardiac myocytes and CPC and decreased cardiac myocyte apoptosis (Khan et al., 2015). This suggests a unique strategy to improve endogenous cardiac repair. Future studies will determine if exosomes from other stem cells types, such as MSC which produce a large number of paracrine signals, can enhance regeneration even more.

## Prospects and conclusions

In summary, the heart has some capacity to regenerate in response to a large insult such as a myocardial infarct, though this process needs to be amplified to repair the tissue. The endogenous regeneration requires an orchestrated participation from various cell types including cardiac myocytes and local and peripheral stem cells. It is naïve to think that only one cell type can participate in this regenerative process and may actually limit therapeutic opportunities.

As discussed in this review, known pharmacological therapies may alter endogenous cardiac regeneration. Future studies are required to expand on this topic to identify treatments that will strike a balance between the drug's primary function and ability to aid or inhibit cardiac regeneration. For example, it may be beneficial to refine the treatments so that they target only specific pathways. A good example of this is in the use of beta blockers. Physicians could choose to treat patients with a beta 1 specific adrenergic blocker, as opposed to a non-specific beta blocker, in combination with a beta 2 specific adrenergic agonist accommodating blockade of the deleterious beta adrenergic signaling (beta 1) and amplifying regenerative downstream effects (beta 2). Statins also appear to be candidates that aid in endogenous cardiac regeneration. Further studies will need to validate this and may identify the mechanisms in which statins can alter endogenous cells, potentially leading to more focused regenerative therapeutic targets. The inhibitory repair effects of drugs such as aspirin may be taken into account in determining a patient's drug regimen.

Refining current pharmacological therapies to enhance endogenous cardiac regeneration has economic and practical benefits for treatment. These drugs are already in current use in clinical practice. As well, generic, low-cost versions of these drugs are available; statin treatment may cost as little as $4 dollars per month (Lazar et al., 2011). Cell and gene therapy approaches are much more expensive and still require approval from regulatory agencies. The costs of preparation and processing of these drugs under good manufacturing procedures is very high. The first gene therapy drug on the market, although for a genetic disease, costs more than $40,000 per vial (Burger and Hirschler, 2014). Clinical administration and multiple treatments augment the price for this type of therapy even more. However, if these therapies can eventually to be proven to heal the heart, then the cost-benefit may be worth the initial price.

To truly improve endogenous cardiac regeneration, the field needs to open itself to the possibility that therapy includes more participants. A multidimensional approach will likely ameliorate the overall repair process, resulting in a more complete regeneration. This is already evident in studies combining both cell and gene therapy where a greater cardiac repair is reported (Schenk et al., 2007; Huang et al., 2014; Ong et al., 2014). One can foresee a multi-faceted treatment plan for patients including pharmacological approaches and cell and gene therapy. This perspective has the ability to translate into other fields of regenerative medicine where endogenous healing may exist, including the central nervous system, diabetes, or bone and cartilage damage.

### Conflict of interest statement

The authors declare that the research was conducted in the absence of any commercial or financial relationships that could be construed as a potential conflict of interest.
